# Long-Term Outcomes Comparison of Endoscopic Resection With Gastrectomy for Treatment of Early Gastric Cancer: A Systematic Review and Meta-Analysis

**DOI:** 10.3389/fonc.2019.00725

**Published:** 2019-08-07

**Authors:** Liangliang An, Sharen Gaowa, Haidong Cheng, Mingxing Hou

**Affiliations:** ^1^Department of General Surgery, Affiliated Hospital of Inner Mongolia Medical University, Hohhot, China; ^2^Department of Pathology, College of Basic Medicine, Inner Mongolia Medical University, Hohhot, China

**Keywords:** endoscopic resection, gastrectomy, recurrence, overall survival, systematic review

## Abstract

**Background:** Endoscopic resection (ER) and gastrectomy have been both accepted as curative treatments for early gastric cancer. We intended to compare ER with gastrectomy treatments on safety of patients, disease-free survival and overall survival for early gastric cancer through this systematic review.

**Methods:** A literature search was performed in Pubmed, Embase, and Cochrane Library databases. Studies that have compared ER with gastrectomy for early gastric cancer were included in this meta-analysis. We searched for clinical studies published before March 2019. Stata 12.0 software was used for systematic analysis.

**Results:** Nine studies were included in this systematic review, ER treatment was associated with a shorter length of stay (WMD = −8.53, 95% CI −11.56 to −5.49), fewer postoperative complications (OR = 0.47, 95% CI 0.34–0.65). ER can be performed safely with shorter hospital stay and fewer postoperative complications than gastrectomy. Recurrence rate was higher for ER than for gastrectomy treatment (HR = 3.56, 95% CI 1.86–6.84), mainly because metachronous gastric cancers developed only in the ER treatment. However, most of the metachronous gastric cancers could be curatively treated with ER again, and it didn't affect overall survival of patients with early gastric cancer. There was no difference in overall survival rate between ER and gastrectomy (HR = 0.84, 95% CI 0.63–1.13).

**Conclusions:** ER and gastrectomy are both acceptable for curative treatment of early gastric cancer. However, due to the comparable overall survival and lower postoperative complications and shorter length of stay, ER is better than gastrectomy for early gastric cancer, who met the indication for ER treatment.

## Introduction

Gastric cancer is one of the most gastrointestinal tract tumors worldwide (1, 2). Even if the incidence of gastric cancer has been declining in the world, it remains one of the most causes of cancer-related mortality in China (3–5). For minimal invasive surgery, the Japanese Gastric Cancer Association's gastric cancer treatment guide lines recommended endoscopic resection (ER) for early gastric cancer (6). ER includes endoscopic mucosal resection (EMR) and endoscopic submucosal dissection (ESD). And, ER is an effective treatment for gastric cancer, but the clinical outcomes of ER in treatment of gastric cancer were controversial (7).

As we know, there were no multi-center studies, which compared the survival benefit between ER and gastrectomy treatments. Only several single-center studies have compared ER with gastrectomy in early gastric cancer (6, 8–15). However, the results of studies were inconsistent. Systematic review and meta-analysis was a powerful and effective method, which could overcome the limitation of small sample sizes of study through combining results from several individual studies, then conduct and achieve a systematic assessment (16). Although, studies comparing ER and gastrectomy in early gastric cancer were most retrospective studies, there is evidence that pooling of high-quality non-randomized comparative studies (NRCTs) is as comparable as pooling randomized comparative studies (RCTs) when assessing clinical surgical outcomes (17). Therefore, we systematically analyzed high-quality clinical researches that have compared ER with gastrectomy in this study and conducted systematic review of combined NRCTs.

The aim of the study was to compare long-term outcomes of ER and gastrectomy treatments for early gastric cancer, and explore whether ER is superior to gastrectomy in early gastric cancer, and we systematically compared length of stay, postoperative complications, disease-free survival and overall survival between ER with gastrectomy treatments in early gastric cancer.

## Methods

### Search Strategy

We conducted and reported this systematic review and meta-analysis following the PRISMA statement (18). The retrieval words are “early gastric cancer,” “early stomach cancer,” “early stomach neoplasm,” “ESD,” “EMR,” “endoscopic resection,” and “gastrectomy.” A search was performed in Pubmed, Embase, and Cochrane Library databases. The studies that have compared ER with gastrectomy for early gastric cancer were included in this meta-analysis. We searched for clinical studies published before March 2019. Meanwhile, we tried to find relevant literature through references of clinical studies. Then we read the full text and determine the eligible studies. Finally, a total of nine studies were included in the analysis.

### Include and Exclude Standards

Studies were acceptable in systematic review if they met these standards: Research compared the outcomes of ER and gastrectomy; Research reported at least one of the following clinical outcomes, including length of stay, postoperative complications, disease-free survival and overall survival; Research was published as a full text in the English language. Research that failed to extract effective data or provide the full text was excluded.

The inclusion criteria of patients: who were newly diagnosed as early gastric cancer, histologically confirmed adenocarcinoma limited to the mucosa or submucosa (TNM stage 0-IIIB), and received gastrectomy or ER for treatment. The exclusion criteria of patients: who had undergone previous gastrectomy. Postoperative pathological evaluation was performed in all included studies. A clear surgical margin was confirmed through pathological evaluation. If a clear surgical margin was not achieved in patients, these patients needed additional ER or gastrectomy. And, patients needed additional gastrectomy were excluded from the study.

### Data Extraction

Two reviewers (Liangliang An, Haidong Cheng) extracted the data of included studies independently and reached consensus on all data. The following data was extracted: authors' name, year of publication, study location, number of patients, length of stay, postoperative complications, disease-free survival and overall survival. HR and 95% CI were used to calculate the disease-free survival and overall survival. Some of the studies included in this meta-analysis provided HR and 95% CI explicitly. If HR and 95% CI were not directly reported in the included studies, we evaluated the HR and 95% CI in the original studies by the methods which illustrated by Parmar et al. (19). Moreover, if the original studies included the median, range and the number of patients, we estimated the mean and variance by the methods illustrated by Hozo et al. (20).

### Assessment of Quality of Included Studies

Quality assessment was peer-reviewed by two reviewers (Liangliang An, Haidong Cheng) independently. Quality scores of the included clinical studies were assessed by the Methodological Index for Nonrandomized Studies (MINORS) (21). We assessed the quality of a study by evaluating 12 items. Studies with ≥18 scores were considered high quality, and were included in the systematic review.

### Statistical Analysis

Systematic review was performed by using statistical Stata 12.0 software (StatCorp, College Station, TX, USA) (22, 23). The test for heterogeneity used the *Q*-test statistic and *I*^2^ statistics. Based on the combined test for heterogeneity, we chose the appropriate method. If there is no heterogeneity among studies (*P* ≥ 0.1), we used the fixed effects model for data consolidation. While there is the heterogeneity (*P* < 0.1) between the results of the study, the random effects model for data analysis would be used. We also explored reasons for inter-study heterogeneity using subgroup analysis by the indication for ER treatment and the endoscopic procedure EMR or ESD. Sensitivity analysis was also conducted by omission of each single study to evaluate stability of the results. Publication bias was evaluated with the Begg's test. A *P*-value of < 0.05 was regarded as significant.

## Results

### Study Selection and Quality Assessment

**Figure 1:**
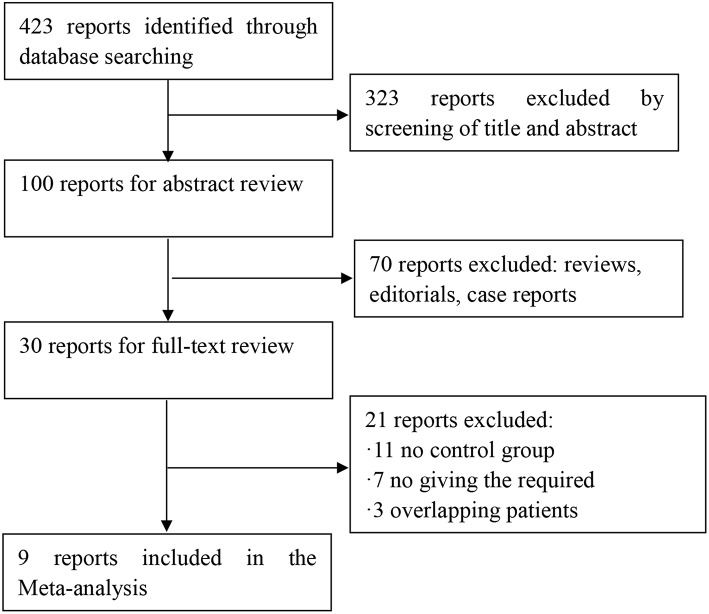
Flow chart for article screening.

Four hundred twenty-three potential articles were generated through our search strategy. After screening the title and abstract, 323 reports were excluded. After reading the research, 70 reports were excluded because they were a review, editorial, or case report. After reading the full text, 11 reports were excluded because there was no control group. Seven were excluded for no giving the required outcomes. Three reports were excluded owing to overlapping patients in multiple studies. The process of our study selection was shown in Figure 1. Nine articles, which were considered to be of high quality, were enrolled in the study. The main characteristics and quality scores of studies are presented in Tables 1, 2.

**Table 1:** Characteristics of studies included in the meta-analysis.

**Study**	**Year**	**Type of study**	**Study period**	**ER indication**	**ER**	**Group**	**Number**	**Age**	**Gender**
Tsuyoshi Etoh	2005	Retrospective study	1085–1999	Absolute indication	EMR(49)	ER Gastrectomy	49 44	84.2 82.2	27/17 31/18
Kwi-Sook Choi	2011	Retrospective analysis with propensity-score matching	1997–2002	Intramucosal gastric cancer	EMR(172)	ER Gastrectomy	172 379	59.3 (9.1) 58.4 (10.3)	127/45 286/93
Philip Chiu	2012	Retrospective cohort study	1993–2010	Mucosal or submucosal involvement	ESD(74)	ER Gastrectomy	74 40	66 (14–88) 67 (33–84)	49/25 23/17
Dae Yong Kim	2014	Retrospective study	2004–2007	Absolute criteria(35) Expanded criteria(107)	ESD(142)	ER Gastrectomy	142 71	62.0 (10.3) 56.7 (12.0)	94/48 58/13
Takeshi Yamashina	2014	Retrospective study	1998–2012	Mucosal or submucosal involvement	EMR(27) ESD(15)	ER Gastrectomy	42 13	71.5 (54–89) 69 (39–76)	40/2 12/1
Ju Choi	2014	Retrospective cohort study	2002–2007	Absolute indication	EMR(86) ESD(175)	ER Gastrectomy	261 114	62 (54–68) 62 (54–66)	195/66 88/26
Chan Park	2014	Retrospectively analyzed the clinical data	2007–2012	Expanded indication	ESD(307)	ER Gastrectomy	307 200	74.5 (3.8) 74.1 (3.5)	211/96 133/67
Young Kim	2014	Prospectively collected clinical data	2001–2009	Expanded indication	EMR(18) ESD(147)	ER Gastrectomy	165 292	62 (54–70) 60 (52–68)	122/43 217/75
Sara Najmeh	2016	Prospectively collected database	2007–2014	Expanded indication	ESD(30)	ER Gastrectomy	30 37	74 (40–86) 75 (34–86)	23/7 24/13

**Table 2:** Quality scores of the included clinical studies were assessed by the Methodological Index for Nonrandomized Studies (MINORS).

**Study**	**A**	**B**	**C**	**D**	**E**	**F**	**G**	**H**	**I**	**J**	**K**	**L**	**Quality scores**
Tsuyoshi Etoh	2	2	1	2	2	2	2	0	2	2	2	1	20
Kwi-Sook Choi	2	2	0	2	1	2	1	0	2	2	2	2	18
Philip Chiu	2	2	1	2	2	2	2	0	2	2	2	2	21
Dae Yong Kim	2	2	1	2	1	2	2	0	2	2	2	1	19
Takeshi Yamashina	2	2	0	2	1	2	2	0	2	2	2	2	19
Ju Choi	2	2	1	2	1	1	2	0	2	2	2	1	18
Chan Park	2	2	2	2	2	2	2	0	2	2	2	2	22
Young Kim	2	2	2	2	1	2	2	1	2	2	2	2	22
Sara Najmeh	2	2	0	2	1	2	2	0	2	2	2	1	18

*A, Clearly stated aim; B, Inclusion of consecutive patients; C, Prospective collection of data; D, Endpoints appropriate to the aim of the study; E, Unbiased assessment of the study endpoint; F, Follow-up period appropriate to the aim of the study; G, Loss to follow up <5%; H, Prospective calculation of the study size; I, An adequate control group; J, Contemporary groups; K, Baseline equivalence of groups; L, Adequate statistical analyses. The items are scored 0 (not reported), 1 (reported but inadequate), or 2 (reported and adequate)*.

### Length of Stay

**Figure 2:**
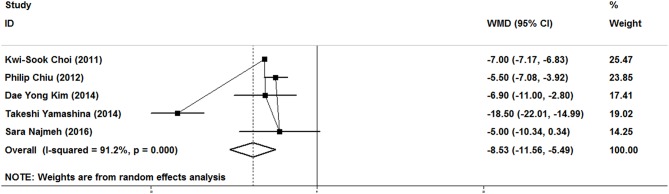
Meta-analysis on length of stay, there was significant difference in length of stay between the ER and gastrectomy treatments.

**Figure 3:**
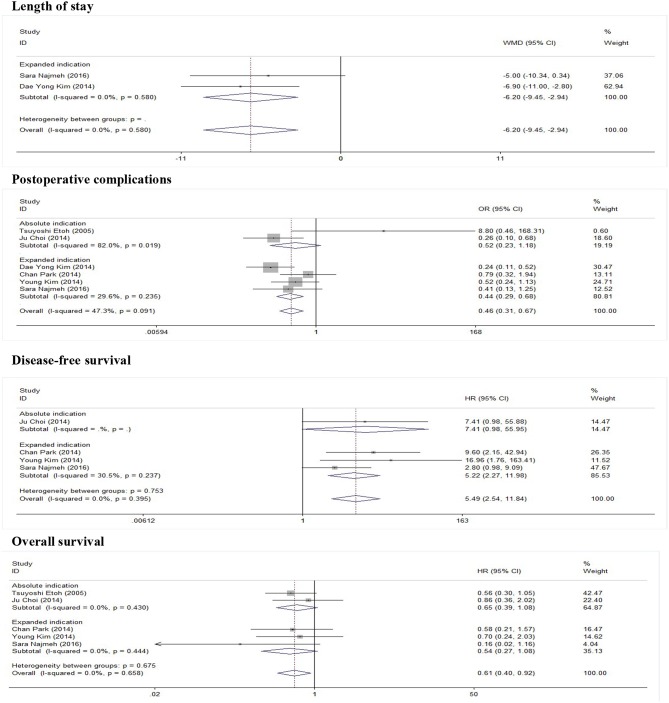
Subgroup meta-analysis of indication for ER treatment.

**Figure 4:**
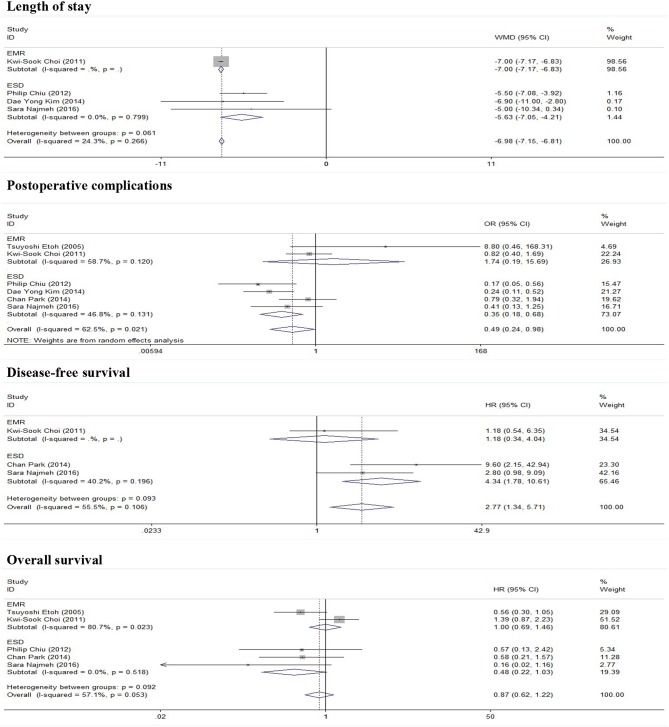
Subgroup meta-analysis of ER procedure.

As show in Figure 2, five studies reported data on the length of stay. Because of significant heterogeneity (*I*^2^ = 91.2%, *P* = 0.000), a random-effect model was used. There was significant difference in length of stay between the ER and gastrectomy treatment for early gastric cancer. ER treatment was associated with shorter length of stay than gastrectomy treatment (WMD = −8.53, 95% CI −11.56 to −5.49). In the subgroup of expanded indication, the difference of length of stay between ER and gastrectomy was also statistically significant (WMD = −6.2, 95% CI −9.45 to −2.94; Figure 3). In the subgroup of ESD, there was also a significant difference in length of stay (WMD = −5.63, 95% CI −7.05 to −4.21; Figure 4).

### Postoperative Complications

**Figure 5:**
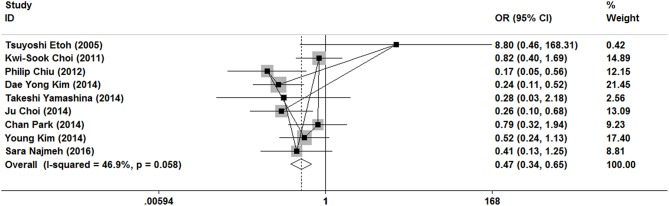
Meta-analysis on postoperative complication, postoperative complications of gastrectomy treatment were higher than that of ER treatment.

As show in Figure 5, all nine researches included postoperative complications. There was no significant heterogeneity (*I*^2^ = 46.9%, *P* = 0.058), and a fixed-effect model was used. The incidence of postoperative complications of gastrectomy treatment were higher than that of ER treatment (OR = 0.47, 95% CI 0.34–0.65). In the subgroup of expanded indication and ESD, there was also a significant difference in complications (Figures 3, 4).

### Disease-Free Survival

**Figure 6:**
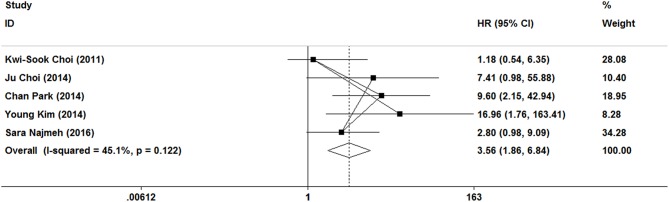
Meta-analysis on disease-free survival, patients who underwent ER treatment had higher recurrence rate than that of gastrectomy treatment.

In this meta-analysis, five studies included the disease-free survival. Because of no significant heterogeneity (*I*^2^ = 45.1%, *P* = 0.122), a fixed-effect model was used. Patients who underwent ER treatment had higher recurrence rate than that of gastrectomy treatment (HR = 3.56, 95% CI 1.86–6.84; Figure 6). The results demonstrated that the recurrence rate of ER treatment was significantly higher than that of gastrectomy treatment. This was most likely because of residual gastric mucosa, which may contain areas at high risk of the development of metachronous gastric cancer. Additional treatments for recurrence lesions should be considered in early gastric cancer patients after ER, but the current studies did not show any adverse event after additional endoscopic treatments for metachronous lesions, and the overall survival of early gastric cancer was no significant difference between ER and gastrectomy. In the subgroup of expanded indication and ESD, there was also a significant difference in disease-free survival between ER and gastrectomy (Figures 3, 4).

### Overall Survival

**Figure 7:**
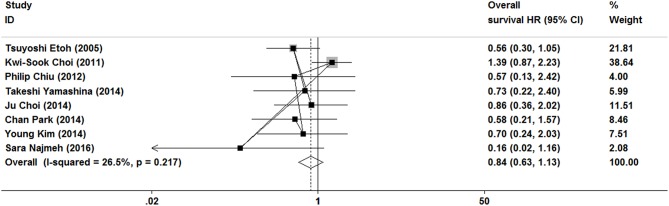
Meta-analysis on overall survival, overall survival did not differ between ER and gastrectomy treatments.

As show in Figure 7, the data of overall survival was reported in eight studies. Because of no significant heterogeneity (*I*^2^ = 26.5%, *P* = 0.217), a fixed-effect model was used. Overall survival did not differ between ER and gastrectomy treatment (HR = 0.84, 95% CI 0.63–1.13). In the subgroup analysis, there was also no significant difference in overall survival (Figures 3, 4).

### Publication Bias

**Figure 8:**
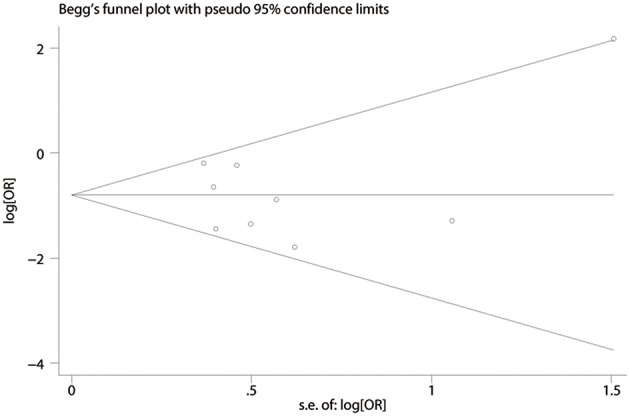
Funnel plot depicting standard error by log relative risk.

**Figure 9:**
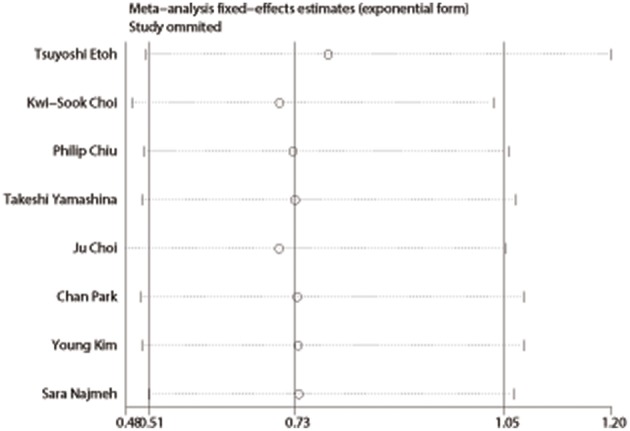
Sensitivity analysis of overall survival.

Publication bias was evaluated based on postoperative complications by using Begg's test. There was no publication bias in nine studies of this meta-analysis (*P* = 0.835). Funnel plot analysis of the studies is shown in Figure 8. Sensitivity analysis also indicated that omitting any single study did not affect the pooled overall survival HR significantly (Figure 9).

## Discussion

In recent years, with the development of digestive endoscopic techniques, more and more early gastric cancer in the absence of any symptoms was found (24, 25). Gastrectomy treatment has been conducted as the conventional treatment for early gastric cancer (26). However, in selected early gastric cancer, ER is accepted due to its minimal invasiveness and better quality of life after the procedure (27). In recent years, ER has become the minimal treatment for early gastric cancer (28–30).

According to the Japanese gastric cancer treatment guidelines, ER includes EMR and ESD (31). And ER is indicated as a standard treatment for the following tumor: a differentiated-type adenocarcinoma without ulcerative findings UL(−), of which the depth of invasion is clinically diagnosed as T1a and the diameter is ≤2 cm. The expanded indication is that Tumors clinically diagnosed as T1a and: (a) of differentiated-type, **UL(−)**, but >2 cm in diameter. (b) of differentiated-type, UL(+), and ≤3 cm in diameter. (c) of undifferentiated-type, UL(−), and ≤2 cm in diameter.

ER was minimally invasive treatment for early gastric cancer, which met guideline or expanded criteria (32). However, clinical outcomes of ER remain controversial, several recent reports suggest that lymph node metastasis may occur after ER treatment in early gastric cancer (33–35). Therefore, treatment outcomes of ER are still controversial for early gastric cancer (36, 37). This meta-analysis combined results from several individual studies to evaluate the outcomes of ER.

In this meta-analysis, a total of nine studies analyzing the ER and gastrectomy treatment were included. This meta-analysis showed that ER treatment showed some advantages, it had a significantly shorter length of stay, and a lower postoperative complication rates. And there were no significant difference between ER and gastrectomy treatments in the overall survival of early gastric cancer. These results of length of stay, postoperative complications, and overall survival were consistent with those of other meta-analyses (38, 39).

There was much evidence to show that the recurrence rate of ER treatment was significantly higher than that of gastrectomy treatment, and the recurrence rates of ER was 4.7–11.1%, and the recurrence rates of gastrectomy was 0.0–1.1%. In this results, the risk of tumor recurrence was significantly higher in the ER group than in the surgery group. This was most likely because of residual gastric mucosa, which may contain areas at high risk of the development of metachronous gastric cancer, such as mucosa with atrophic gastritis and intestinal metaplasia (40). Additional treatments for recurrence lesions should be considered in early gastric cancer patients after ER, but the current studies did not show any adverse event after additional endoscopic treatments for metachronous lesions, and the overall survival of early gastric cancer was no significant difference between ER and gastrectomy treatment. And, metachronous gastric cancer did not affect overall survival (6, 11, 15).

There are some limitations of this meta-analysis. The approach of extrapolating the HR of overall survival was a potential factor might lead to heterogeneity of outcomes. Moreover, this meta-analysis only included fully published studies. Unpublished researches were not included in meta-analysis. In addition, this study was searched with language restriction, so this analysis only included studies in English.

In conclusion, ER and gastrectomy are both acceptable for curative treatments of early gastric cancer. However, ER is better than gastrectomy for early gastric cancer, who met the indication for ER treatment, due to the comparable overall survival and lower postoperative complications and shorter hospital stay.

## Data Availability

The raw data supporting the conclusions of this manuscript will be made available by the authors, without undue reservation, to any qualified researcher.

## Author Contributions

LA and SG: development of methodology. SG and HC: acquisition of data (acquired and managed patients, provided facilities, etc.). LA, SG, and HC: analysis and interpretation of data (e.g., statistical analysis, computational analysis). HC and MH: writing, review, and/or revision of the manuscript. MH: study supervision.

### Conflict of Interest Statement

The authors declare that the research was conducted in the absence of any commercial or financial relationships that could be construed as a potential conflict of interest.
